# Construction of a Knowledge Map of Speech Emotion Features Based on Impulse-Coupled Neural Networks

**DOI:** 10.1155/2022/8766145

**Published:** 2022-07-08

**Authors:** Yan Song

**Affiliations:** Henan Polytechnic, Zhengzhou, Henan 450046, China

## Abstract

This paper constructs knowledge graphs of speech emotion feature to support the expression of rich semantic information due to their unique graphical structure and to provide new ideas for studying speech emotion recognition recommendations. Impulse-coupled neural networks, as a mathematical abstraction of the visual properties of the human eye, have been widely used in various fields of speech processing. In this paper, we take the classical impulse-coupled neural network model as the research object, aiming to explore, analyze, and study the method of constructing knowledge maps of speech emotion features based on the impulse-coupled neural network model, and propose several improved impulse-coupled neural network models, which are used in the fields of target detection, speech segmentation, and quantization compression. This paper further constructs various domain semantic concept maps and performs multidimensional semantic enhancement understanding of search text based on the concept maps and the constructed entity-relationship knowledge maps and proposes a knowledge-based interpretable recommendation method for cloud services and a generalized recommendation and sample enhancement method for cloud scenarios. The fusion algorithm based on multiscale analysis can decompose the source speech into subspeech at different scales and then fuse each subspeech separately, while typical objects in the real world are also composed of many components at different scales. Based on the factors of different degrees in the speech context, the imagery work ruler evaluation model is refined with the basic principles of risk perception and risk resolution in the context, and an exploratory design is carried out separately for the verbal description, graphics, text, color, animation, and sound signals in the audiovisual signal using the workshop format to obtain a large number of design samples and evaluate the important units in turn, finally integrating the context involved in the study. The perceptual design system is given as a specific contextual design method.

## 1. Introduction

In today's era of information explosion, personalized recommendation systems based on speech emotion recognition are the core technology for user-oriented Internet products. Recommendation systems can help users get the information they need and improve the problem of information overload. The technical core of a recommendation system is to model information such as user history, item attributes, and context; infer the user's interests; and recommend items of interest to the user. Therefore, practical recommendation algorithms need to be highly scalable and can easily incorporate various auxiliary information [1]. Among the many auxiliary information, there is a special class of information that possesses a network structure, for example, online social networks among users, knowledge graphs among items, or even the interaction between users and items itself constitute an interaction graph. Network-structured information provides a rich auxiliary input for recommendation algorithms, yet how to effectively utilize such high-dimensional structured data becomes a challenging problem in recommendation systems.

Network feature learning is gradually becoming a popular research direction in machine learning. Network feature learning attempts to learn a low-dimensional representation vector for each node in a network while maintaining its original structural information [2]. There are a large number of network structures in the recommender system. Combining the network feature learning with the recommender system and using the network feature learning method to process the relevant features in the recommender system can effectively enhance the learning ability of the recommender system. This will improve the accuracy and user satisfaction of the recommender system, thus providing a better user experience for various Internet applications in real life. The negative impact of the information explosion and the overall economic efficiency can be improved.

Given that current recommendation systems mostly use collaborative filtering-based, content-based, and knowledge-based algorithms, they largely do not take into account the multilayer relationship between products and products. The algorithm does not have good performance for all scenes of source speech and lacks stability and flexibility. Moreover, since this algorithm is a pixel-level fusion, incomplete fusion conditions such as source speech containing noise or not fully aligned will have a great negative impact on the results. This project will explore the construction method of a knowledge graph of speech emotion features in the cloud service domain based on Pulse-Coupled Neural Network (PCNN) and contribute to the construction of a domain knowledge graph. Based on the established domain knowledge graph, a cloud service recommendation system is constructed [3]. The system can have the function of cloud knowledge adaptive learning, and at the same time, it can realize the cloud service recommendation based on the user's scenario demand based on natural language processing and knowledge graph inference according to the user's demand.

Pulse-coupled neural networks are two-dimensional neuronal networks formed by the interconnection of several neurons, which can well simulate the properties of biological neurons, where neurons with similar inputs can be triggered (also called ignition) simultaneously under the simultaneous action of forced and evoked stimuli. When applying the impulse neural network to speech feature processing, this two-dimensional neuron network classifies speech feature points based on spatial and luminance similarity. Multiscale analysis-based fusion algorithms can decompose the source speech into subspeech at different scales and then fuse each subspeech separately, while typical objects in the real world are composed of many components at different scales, so these algorithms can represent the inherent features of speech well, which is helpful to improve the quality of fused speech [4]. Conventional fusion algorithms based on multiscale analysis have predefined transformations, which do not have a good representation of source speech in all scenes and lack stability and flexibility, and because these algorithms are pixel-level fusion, incomplete fusion conditions such as source speech containing noise or not fully aligned can have a great negative impact on the results, and how to overcome the problem of fusion quality while ensuring and how to overcome the low efficiency of general feature-level algorithms and the difficulty of balancing quality and efficiency are the problems that need to be solved at present.

The PCNN model is highly valued in the field of feature recognition because of its spatial proximity and similar clustering of pixel gray values to issue pulses, which can well detect the local location information of features. In the literature [5], it was reported that synchronized pulses could occur by stimulating the cerebral cortex of small mammals, and after an in-depth study, a connectivity model with pulse-emitting properties was proposed. In the literature [6], it was found that when using the above connectivity model to process features, not only could it retain the periodic pulse-issuing characteristics of the model but also its processing results would not be affected by the change of features, a simplification and modification scheme of the model was proposed, and the pulse-coupled neural network model, i.e., the traditional PCNN model, was formally proposed. However, the traditional PCNN model is too complex in its operation when applied to feature segmentation, and the parameter settings are highly subjective, so some scholars have made a series of attempts to improve the PCNN model feature segmentation method by simplifying the model and combining other algorithms and have achieved certain results. The literature [7] proposed to use of the maximum entropy criterion as the criterion to determine the best result of PCNN feature segmentation and achieved the automatic termination iteration of the PCNN model. The principle is that after inputting the target features into the PCNN model, the maximum entropy is calculated for the segmentation results of each iteration, and the output features are terminated when the maximum entropy no longer changes in *N* iterations or changes less than the set value, which is the feature segmentation result containing the maximum amount of feature information. This method reduces the experimental process of the number of iterations of the PCNN model and reduces the interference of human subjective judgment on the segmentation results, which is of great significance in the field of PCNN feature segmentation. The literature [8] proposes to use the minimum cross-entropy criterion to implement the number of iterations of the PCNN feature segmentation algorithm and to realize the automatic selection of the optimal threshold for feature segmentation. The principle is to combine the cross-entropy algorithm with the PCNN algorithm to automate the setting of the number of iterations of the PCNN feature segmentation algorithm by judging the difference in the amount of information contained between the original features and the segmentation results and selecting the segmentation result with the minimum cross-entropy as the termination condition. The literature [9] combines the simplified PCNN model with a genetic algorithm, which enhances the algorithm's ability to solve random searches and also makes the PCNN feature segmentation method with self-contained bio-visual characteristics have a stronger biomimetic ability, which not only realizes the automatic segmentation of features but also automates the setting of key parameters of the PCNN model. The principle is to convert the connection coefficients, magnitude coefficients, and attenuation coefficients in the PCNN model to binary combinations into chromosomes in genetic algorithms; perform crossover, mutation, and selection operations; select the best parameter values using the maximum Shannon entropy as the fitness objective function; and finally use the maximum entropy as the criterion for determining the superiority of segmentation results to achieve automated termination iterations. Anbalagan et al. [10] tried to use an adaptive algorithm of the grayscale-information histogram to achieve the r-motivated setting of the temporal decay coefficient of the PCNN model, and the simulation results show that the method can effectively reduce the number of iterations of the PCNN model while completing feature segmentation, thus reducing the overall algorithm's computing power, and the method can solve the problem of segmentation of multiple target markers when the background information is very complex. However, the method only sets the time decay coefficient of the PCNN model, and other model parameters still need to be set by manual experiment. The literature [11] proposed that when performing PCNN feature segmentation, the Tsallis entropy of the target feature gray value histogram can be calculated first, and the two-dimensional Tsallis entropy is used as the judgment criterion of the feature segmentation results to realize the automated termination of PCNN feature segmentation, and the method has a corrective effect on the decay rate of the dynamic threshold term of the PCNN model.

The literature [12] experimented with the automated setting of the connection coefficients of PCNN models by performing bilinear interpolation operations on the features and then determining the connection coefficients based on the anisotropic diffusion characteristics of the features, which not only achieved setting the connection coefficients of PCNN models with objective algorithms but also enabled the segmentation results to retain more contour information and edge details. In the literature [13], an automatic parameter setting method for simplified impulse-coupled neural networks was proposed. The method successfully determines all adjustable parameters in the SPCNN and does not require any training and experimentation as required by previous methods. In the literature [14], the GIT-PCNN, an impulse-coupled neural network model based on grayscale iterative thresholding, is proposed to replace the traditional PCNN model with exponentially decaying thresholds with grayscale iteration-based feature thresholds. The grayscale information of the features is cleverly combined with the local area information of the features excited by the unique synchronous pulse release feature of PCNN, and the feature segmentation is completed in one firing without selecting the network parameters and setting the termination cycle conditions. The literature [15] analyzes the mathematical characteristics of the model itself from the iterative equations of the PCNN model, reveals that the PCNN model itself generates disturbances and effects, analyzes the generation mechanism and elimination methods, and proposes an adaptive improvement setting method for the PCNN parameters and improves the model with good results.

Pulse-coupled neural networks have shown unique advantages in feature segmentation, but there are still shortcomings in their development. The main problem is that PCNNs have complex network structures and more parameters, and whenever the object changes, the network model parameters need to be readjusted to obtain satisfactory results. Up to now, this problem is still solved mainly by manually selecting parameters through multiple experiments, which greatly limits the popularization and application of PCNN. Therefore, the in-depth study of PCNN network parameter selection methods is still a hot spot and difficult area in the field of feature processing in the future.

## 2. Exploring the Application of Impulse-Coupled Neural Networks in Building Knowledge Graphs of Speech Emotion Features

### 2.1. Modeling of Impulse-Coupled Neural Networks

The pulse-coupled neural network is based on synchronous dynamics to mimic the neuronal activity of the cat's visual cortex. More importantly, compared with traditional multilayer feedforward and convolutional neural networks, pulse-coupled neural networks are neural networks consisting of dynamic neurons with a single layer of two-dimensional locally connected structures that do not require training [16]. In the process of designing and building the knowledge graph, the first step is to design the main structure of the speech emotion feature recognition business, which can be used quickly and also provide convenience for later operation and maintenance; the second step is to carry out data collection and entry, collect entities with practical meaning by manual and automated means, and carry out preliminary data cleaning; after getting a cleaner data set, all instances that appear many times in the data set and represent the same object are fused with knowledge, and entities with less information and less meaning are deleted based on a priori knowledge. The impulse-coupled neural network model is researched and developed based on the visual characteristics of mammals and is widely used in computer vision.

**Figure 1:**
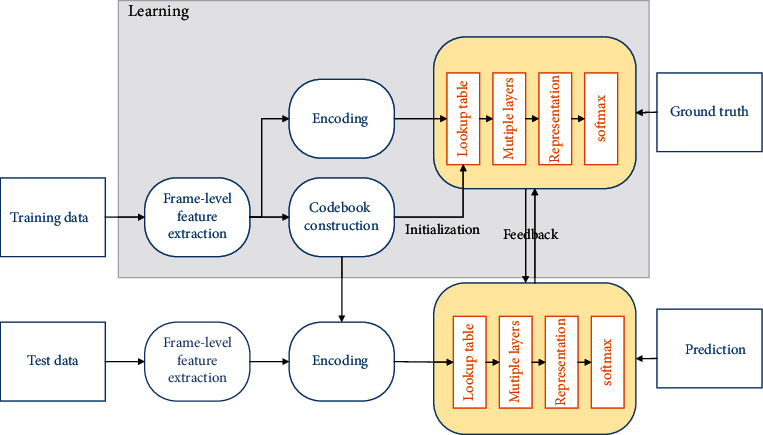
A framework of the impulse-coupled neural network model.

A PCNN neuron contains two main components, as shown in Figure 1, the feed-in input and the connection input, which are connected to adjacent neurons via synaptic weights *K* and *L*, respectively, and retain the previous state with the presence of attenuation factors *e*^−*α*^ and *e*^−*β*^, respectively, and only the feed-in input *K*_*ij*_[*n*] receives external input stimuli *M*_*ij*_:

(1) $$\begin{array}{c} K_{ij}\left[n\right]=e^{-\alpha }K_{ij}\left[n-1\right]+e^{-\beta }\sum_{i=1,j=1}^{n}M_{ij}\cdot Y\left[n-i\right]\cdot X\left[n-j\right], \end{array}$$

where *e*^−*α*^ and *e*^−*β*^ are the scaling coefficients of the feed-in and connection inputs, respectively, *n* is the number of iterations, and *Y*[*n* − *i*]*X*[*n* − *j*] represent the positions of different neurons. The feed-in and connection inputs are modulated by the connection strength to obtain the internal activity *R*_*k*_. The internal activity *R*_*k*_ is compared with the dynamic threshold *f* to produce the final output value.

(2) $$\begin{array}{c} R_{k}=\sum_{i=1}^{k}f_{\text{k}}\left(w_{k}\right). \end{array}$$

If the neuron ignites, i.e.,

(3) $$\begin{array}{c} M_{ij}=0. \end{array}$$

The threshold value will then increase because of the influence of the amplitude *w*_*k*_, thus making the neuron less likely to ignite successfully; if the neuron ignites unsuccessfully, the amplitude *w*_*k*_ will not affect the threshold size, and the threshold value will then decay continuously because of the influence of the factor, thus making the neuron more likely to ignite. As the feed-in channel, which affects the transmission distance of the automatic wave generated by the coupled pulse network, which affects the decay rate of the feed-in input, the weight matrix directly affects the transmission speed of the automatic wave, and its value determines the strength of the synaptic weights.

**Figure 2:**
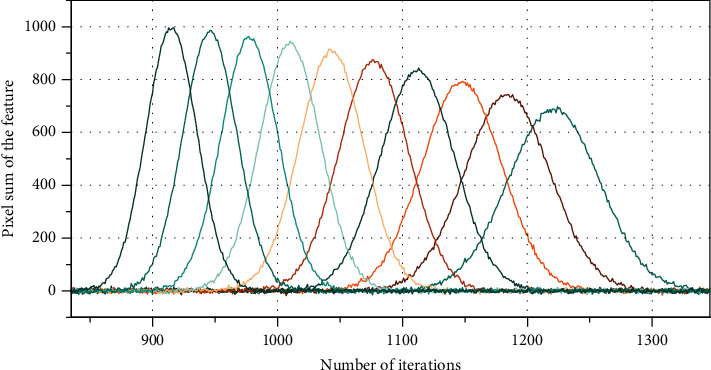
Periodic table of the pulse-coupled neural network.

There is a local excitation spreading mechanism in pulse-coupled neural networks, and when a neuron produces excitation, it transmits this excitation to the surrounding neurons, causing excitation in neurons that are otherwise in an inhibited state [17]. However, this excitation is limited, and the excitation generated by the excitation of surrounding neurons does not persist. Because of the self-inhibitory nature of pulse-coupled neural networks, the excitation of each neuron is time-limited. For example, when a person receives a mosquito bite, the pain is felt in the fingers and transmitted to the brain, but not to the feet or the lower back or the ears, etc. This is an example of the local excitation spreading mechanism of the pulse-coupled neural network. Therefore, based on this bionic structure, the pulse-coupled neural network also inherits some of these properties. For the kind of neural network, different pulse-coupled neural networks differ only in setting different parameters to the network, which determines some of the characteristics of the network. The periodicity of the impulse-coupled neural network is easily observed when a feature is decomposed at multiple scales. The multiscale decomposition of the feature is first performed, and then, the output results of each pulse-coupled neural network in the feature are accumulated separately to calculate the pixel value of the current feature and generate the number of iterations with the pixel sum of the feature as in Figure 2.

The periodicity of the impulse-coupled neural network is investigated from different observation angles: periodicity of pulse-coupled neural networks on the same source feature, on the same scale; periodicity of pulse-coupled neural networks on the same source feature, on a different scale; and periodicity on different features, on any scale, in any shear wave direction.

To simplify the problem and eliminate the interactions between multiple neurons, the range of pixel bands with different time ignitions for the single neural case is first solved. Because the feed-in input is larger than the dynamic threshold, the neuron ignites and this ignition is due to the initial value of the neuron [18]. For the feed-in channel, which affects the transmission distance of the automatic wave generated by the coupled pulse network and the decay rate of the feed-in input, the weight matrix directly affects the transmission speed of the automatic wave, and its value determines the strength of the synaptic weights. For the convenience of later description, this time ignition of the neuron due to the initial value problem is ignored and the next ignition of the neuron is the initial ignition by default. At the *n*th iteration, the

(4) $$\begin{array}{c} K_{nn}=e^{-\alpha }K^{\prime }+e^{-\beta }M\left(x,y\right)\cdot X_{nn}\cdot Y_{nn}. \end{array}$$

If the ignition is successful, then

(5) $$\begin{array}{c} K_{nn}=\sum_{i=1}^{k}h_{k}\left(v_{k}\right). \end{array}$$

If ignition fails, then

(6) $$\begin{array}{c} K_{nn}=R_{\text{k}}\cdot L_{k}^{2}+\int Q_{k}x\text{d}x. \end{array}$$

Therefore, it can be deduced that to achieve the initial ignition at the *n*th iteration, the condition needs to be satisfied that

(7) $$\begin{array}{c} M\left(x,y\right)=\left\{\begin{array}{cc} M_{ij}\times x+\varepsilon \text{free}\times x^{2}\times d^{1/2}, & d\leq d_{0},\\ M_{ij}\times x+\varepsilon \text{decay}\times x^{2}\times d^{2}, & d>d_{0}. \end{array}\right. \end{array}$$

To verify the correctness of the formula, the results of the speech-like processing under this algorithm are used for verification, but the law is not limited to parameter-adaptive impulse-coupled neural networks. Because the difference between parameter-adaptive impulse-coupled neural networks and conventional impulse-coupled neural networks is only whether the parameters are adaptive or not, it is essentially the network parameters that determine the processing effect, and the formula is essentially determined by the parameters of the network, so it does not affect its correctness [19]. Another point to note is that for the speech samples, the larger the pixel band is, the more valuable the pixel value tends to be, i.e., the earlier the initial ignition of the neuron is achieved, the more important the information it represents, and as long as these pixel bands are calculated correctly, then the derived formula is valuable.

Based on this result, it can also be deduced that assuming that a neuron achieves initial ignition at the kth iteration, the time difference between its next ignition time and the current ignition time is *l*. Therefore, it can be concluded that the range of pixel bands that achieve ignition at each time point is determined at the initial ignition, and does not vary much. Again, it should be noted that the law highlighted above is for the vast majority of pixel points, and there is a small percentage of pixel points that do not obey such a law. Because the law of PCNN is that a certain nerve ignition affects the surrounding nerves, the current PCNNs are all stimulatory effects and no inhibitory ones [20]. That is, a certain nerve ignition may make the surrounding nerves ignite earlier, but this transmission will not be unrestricted.

### 2.2. Knowledge Mapping for Speech Emotion Feature Recognition Based on Impulse-Coupled Neural Networks

**Figure 3:**
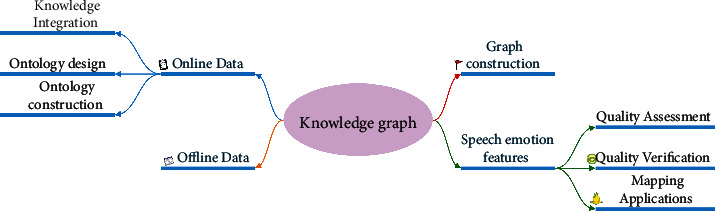
Knowledge graph construction process of speech emotion features.

Knowledge graphs are usually constructed in two ways: top-down and bottom-up. Since the information to be collected comes from a wide range of sources, each data source needs to be treated with a targeted approach to extract useful information from it. The paper adopts a top-down approach to construct a knowledge graph of speech emotion features in four steps: construction of speech emotion features ontology, knowledge extraction, knowledge fusion, and knowledge storage as shown in Figure 3.

In the process of designing and constructing the knowledge graph, firstly, the main structure of the speech emotion feature recognition business needs to be designed, which can be used quickly and also provide convenience for later operation and maintenance; the second step is to carry out data collection and entry, collect entities with practical meaning by manual and automated means, and carry out preliminary data cleaning [21]. After obtaining a relatively clean data set, all the instances that appear several times in the data set and represent the same object are fused with knowledge, and the entities with less information and less significance are removed based on a priori knowledge; finally, all the entities are constructed into a knowledge graph, which is also randomly sampled and manually annotated to evaluate the accuracy and reliability of the knowledge graph.

In the process of knowledge fusion, entity alignment is needed to determine whether two or more entities from different information sources are the same entity. Due to the difference between official and unofficial expressions, there are differences in the representation of the same entities, which is the main and important problem faced in the knowledge fusion phase, and therefore, the entity names need to be standardized. In the process of knowledge fusion, it is necessary to map certain strings in a piece of text to corresponding entities in the knowledge base through entity links to eliminate consistency problems such as entity conflicts and unclear pointers. Finally from the knowledge graph used to provide knowledge support to the Q&A system, the data need to be stored in the database after knowledge fusion. For storing such a semantic network as a knowledge graph, a graph database in the form of a “network data structure” is more suitable as the carrier of the knowledge graph. The paper adopts a popular open-source graph database, Neo4j, for storing knowledge graphs, which is characterized by its compliance with knowledge graph storage logic and high-quality visualization of knowledge graphs.

Information ecology refers to the mutual relationship between information people and their surrounding information environment, that is, it involves the mutual influence and interaction between the information environment, information people, information, and information technology. Information ecology is the use of ecological views and methods, comprehensive consideration of the relationship between various elements, and the reasonable deployment of resources in the information ecosystem, to maintain the information ecosystem calm, stable, and orderly. The information ecosystem mainly includes the following connotations: (1) information ecosystem is an organic part of the large social system and is a dynamic system with diversity and complexity. An information ecosystem is a harmonious, dynamic, and balanced self-organized system composed of four parts: information subject, information object, information environment, and information technology. (2) The main body of the information ecosystem is the information person, which refers to the group of individuals or organizations involved in information activities, consisting of information producers, information disseminators, information organizers, information consumers, and information decomposers, and has the dynamic nature of role transformation. (3) The object of the information ecosystem is information, which is the basic element to maintain the operation and development of the whole information ecosystem and is the connecting link between information people and the information environment. (4) Information ecosystem environment refers to the collection of factors outside the information subject that have a direct or indirect influence on the survival and development of the information subject. (5) Information ecological technology is the key to supporting the existence of an information ecosystem and is the key technology for information subjects to visualize information objects in the information ecosystem environment, mainly including information storage, transmission, and processing [22].

Information ecological chain research originates from the cross-application research of information ecology and information chain. An information ecological chain is an artificial system in a specific ecosystem, which concentrates the constituent elements of information ecology such as information environment, information people, information, and information technology. Information is processed and transmitted in the information ecological chain in a way similar to the operation of an assembly line and participates in the flow of the whole ecosystem. The information subjects (information people) in the ecosystem are arranged in a chain-like structure according to the order of information flow. The basic structure of the information ecosystem includes information person, information, and dissemination path. In the information ecological chain, the information subject (information person) is the core element, and the development direction of the information ecological chain is influenced by the information subject; information is the key factor in the formation of the information ecological chain; information environment is the sum of various spatial factors for the survival of the information subject, and it is the supporting background for the existence of the information ecological chain; information technology is a single influence factor independent of the information environment, and it is the supporting condition for the optimization of the development of the information ecological chain. The periodic pulse-coupled neural network on the same source feature and the same scale has different periodicity on the same source feature at different scales. In the information ecological chain, the information subject is supported by information technology, and the information subject and the information environment are constantly connected and interactively influenced by the flow of information [23]. In the information environment, the information subject forms a chain dependency relationship through the process of production, dissemination, organization, consumption, and decomposition of information through information technology. The coordinated development of each information ecological factor promotes the continuous evolution of the information ecological chain.

**Figure 4:**
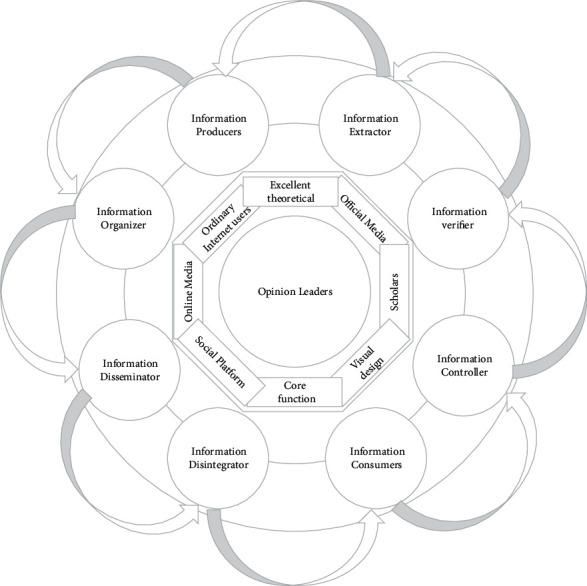
Topic element composition of the knowledge map of speech emotion features.

The information on speech emotion features in the information ecological chain is collected through a pulse-coupled neural network and combined into a knowledge map of speech emotion features, whose subject element composition is shown in Figure 4. There are two connotations of information resources: one is as the glue of information subjects in the information environment to maintain or promote the connection or role between each subject and the other is as the processing object in the whole information environment, acting as the basic element for the operation of the whole speech emotion characteristic information environment. Information people's demand for information determines information resources, and information resources, as the medium between information people, are also influencing information people's demand for information. In other words, information resources as a whole reflect a certain value preference of information people in the information environment, and the value preference already formed in the information environment also influences the information people in the information environment to a certain extent. The subject elements of the knowledge map of speech emotion characteristics correspond to information people in the information ecosystem, which refer to the opinion users who use corresponding information technology to express their views, emotions, opinions, and attitudes by releasing opinion information under a certain space of speech emotion characteristics. Information people include ordinary Internet users who account for the majority of the voice emotion feature space, key node users who play the role of key links, opinion leaders who have a dominant position in the voice emotion feature, and control subjects who guide the voice emotion feature. These kinds of information people play different information roles in the information ecology.

In the speech emotion trait knowledge mapping, information environment, information person, information, and information technology together constitute the information ecosystem of speech emotion trait knowledge mapping. The environment element, subject element, object element, and technology element together serve as the four major information ecological elements of speech emotion characteristic user topic mapping. The process of knowledge fusion requires mapping certain strings in a piece of text to the corresponding entities in the knowledge base through entity links to eliminate consistency problems such as entity conflicts and unclear pointers. Finally, from the knowledge graph used to provide knowledge support to the Q&A system, the data need to be stored in the database after knowledge fusion. The construction of user topic mapping of speech emotion features relies on mature knowledge mapping processing technology, which mainly includes entity recognition, attribute extraction, relationship extraction, and topic mapping built on the corresponding model. Among them, entity recognition is the support for the topic mapping of speech emotion feature users, and through entity recognition, the nodes constituting the topic mapping can be accurately located from a large number of speech emotion feature users. Attribute extraction is an important component of the topic mapping of speech emotion feature users, which helps to describe the nodes in the topic mapping with accurate attributes. Relationship extraction is the key to building the topic mapping of speech emotion feature users, and the relationship between nodes determines the connection between different nodes of the topic mapping and the mechanism of action. The construction of the topic mapping lies in selecting the appropriate model to identify and extract the node types or edge relationships in the mapping.

### 2.3. Experimental Design

To verify the validity of the model, the following experimental contents and processes are designed in this chapter: (1) training/testing set division. Each experiment randomly selects 80% of the data set as the training set and the remaining 20% as the test set, which is not involved in training and is only used to verify the model performance; (2) training and selection of corpus vectors. To ensure the quality of the corpus vectors, a corpus of news data from the whole network is used to train the word vectors. The corpus collected more than 20,000 real news data from several news sites, covering 18 channels of society, sports, news, entertainment, etc., which involved many fields, and the amount of data was very sufficient. The corpus was selected for word vector training to enhance the generalization ability of the model by enhancing the generalization type of the word vector; (3) constructing a convolutional neural network model. The Keras deep learning toolkit is used, and TensorFlow is chosen as the underlying implementation; (4) hyperparameter tuning. Considering the influence of hyperparameters on the model performance, the convolutional kernel size, activation function, dropout random deactivation ratio, and the number of iterations are adjusted; (5) comparing the experimental effects.

The initial sentiment text is obtained after data collection and preprocessing, and it needs to be vectorized into computable numerical data. In this paper, we use word embedding to map each word into a low-dimensional vector, which further represents the sentiment text into a high-dimensional matrix stitched by the word vectors. The Word2Vec model is used in the research process to map each word or word after the word separation process into a vector, and the cosine similarity is used as a metric to evaluate the word-level vectors as shown in the following equation:

(8) $$\begin{array}{c} G=f^{P}\left(i\right)⊕f^{Q}\left(j\right)=f\left(P_{i}\right)f^{Q}{\left(j\right)}^{T}. \end{array}$$

The tail of the whole model is two fully connected layers. The first fully connected layer is 128 neurons, and the second fully connected layer is 64 neurons so that the text matrix after feature extraction can be restretched into a one-dimensional vector. The dropout layer is added to control the number of randomly deactivated neurons in the fully connected layer because the use of fully connected layers can greatly increase the number of parameters in the model and even cause overfitting, and the dropout randomly designates some neurons to have zero output in each iteration so that their connected weights will not participate in the weight update during the back-propagation training process. This controls the number of parameters of the model and facilitates the computation on the one hand; on the other hand, it also cuts the nonessential dependence among the weights, further controls the risk of model overfitting, and significantly improves the performance of the model on the test set.

## 3. Results and Analysis

### 3.1. Regularized Parameter Analysis

To select the appropriate regularization parameters, seven sets of controlled experiments were conducted. The experiments were conducted mainly between multifocused samples, which represent the case where the information of the sample is spread over the whole sample, and regular samples, which represent the case where the information of the sample is concentrated in the center of the feature. Therefore, these two types of samples can be chosen to test the appropriate values of the regularization parameters in different situations.

**Figure 5:**
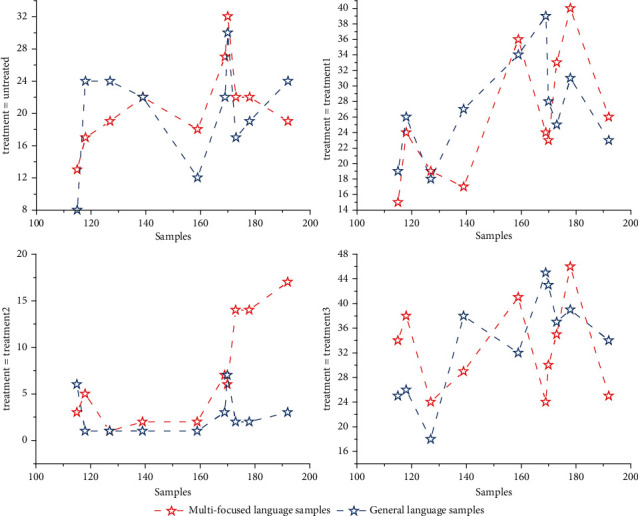
Effect of regularization parameters on objective performance.

As shown in Figure 5, different values of the regularization parameter *γ* are different in multifocused and regular speech samples. The appropriate value of *γ* can protect the boundary information of the speech samples, and the multifocused speech samples with dispersed speech information are more suitable for larger *γ* values, while the regular speech samples with more concentrated speech information are more suitable for smaller *γ* values. Therefore, based on the above observations, *γ* is set to 5 for multifocused samples representing a class with scattered discourse information, while *γ* is set to 0.01 for regular samples representing a class with more concentrated discourse information. firstly, the user input questions and templates are divided into words, the frequency of each word is counted, and the value of inverse document word frequency is calculated. When there are *N* entries, the template and input data can be represented by an *N*-dimensional vector, and the value on the dimension indicates the IDF value of the word on the input data or module. The cosine value of all the templates and the input data is calculated. A large cosine value indicates that the corresponding vector angle is smaller, and at the same time, the two are more similar, and a threshold is set, and the template with a cosine value greater than the threshold can be used as the answer. When multiple templates are similar, the template with the largest cosine value is taken as the user's intention, and the corresponding entity relationship is found from the Neo4j repository to make the answer.

**Figure 6:**
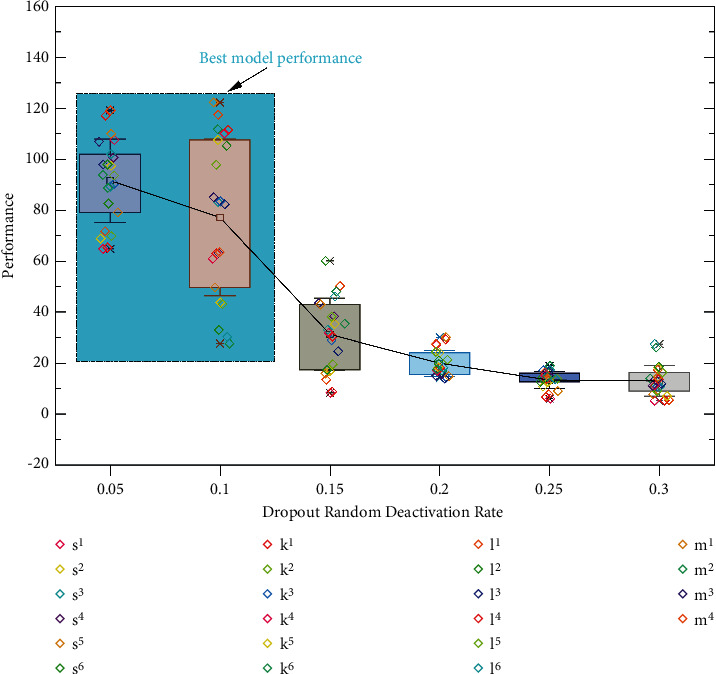
Effect of dropout random deactivation rate on model performance.

The random deactivation rate of the dropout layer and the number of training iterations are also key factors affecting the model performance. The following is the validation process of the relevant experiments: firstly, the impact of the dropout random deactivation rate on the model performance is tested, and the relevant indexes on the test set are shown in Figure 6, which shows the trend of the impact of dropout random deactivation rate on the model. When the dropout random deactivation rate is in the range of 0.05–0.1, the indicators of the model reach the optimal effect; when the random deactivation rate exceeds 0.1, the overall indicators of the model show a decreasing trend. Therefore, the best model performance can be obtained by setting the dropout random deactivation rate between 0.05 and 0.1.

### 3.2. Validation of the Effect of Hyperparameters on the Model

**Figure 7:**
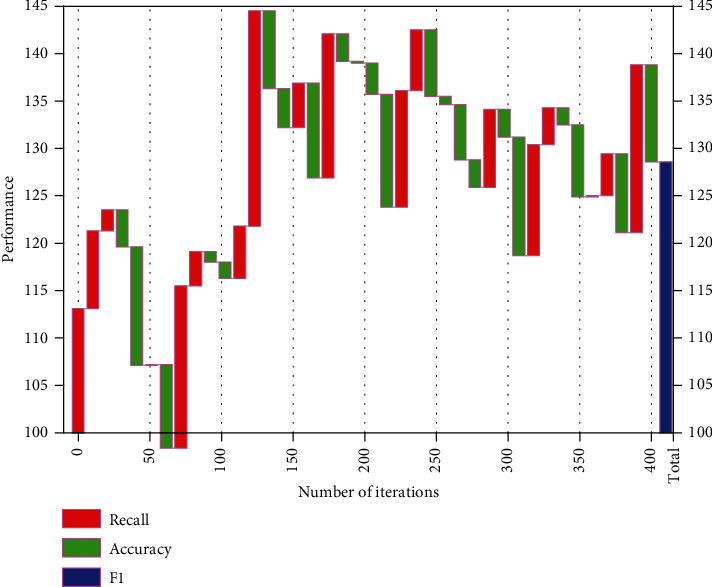
Effect of the number of iterations on the relevant indexes.

The number of iterations in pulse-coupled neural networks often depends on the specific task type. Compared with other hyperparameters, the number of iterations directly affects the training time of the model and influences the final fit. If the number of iterations is too small, the model will not be able to converge to the local minima and lead to underfitting; conversely, if the number of iterations is too large, it will increase the time cost of model training on the one hand and lead to overfitting problems on the other hand, which will reduce the generalization ability of the model. Information environment is the sum of various spatial factors for the survival of the information subject, and it is the supporting background for the existence of the information ecological chain; information technology is a single influence factor independent of the information environment, and it is the supporting condition for the optimization of the development of the information ecological chain.  Figure 7 shows the changes in the model-related indexes when the number of iterations is 0–400, respectively. From Figure 7, it can be seen that after 15 iterations, the model indicators reach their peak, but after 16 iterations, the indicators have a significant downward trend, indicating that the model has been overfitted.

**Figure 8:**
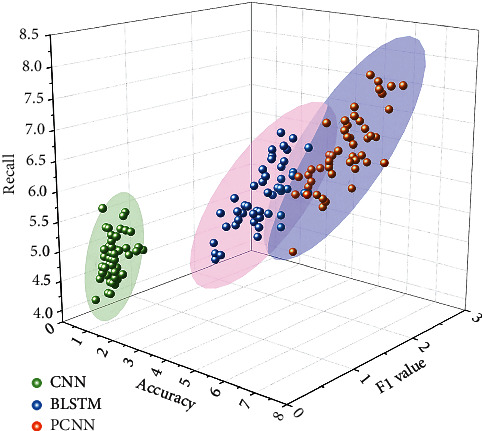
The recognition accuracy of the PCNN method and comparison algorithm in various types of relationships.

As shown in Figure 8, the performance index of the PCNN algorithm in the extraction of entity relationships in scientific and technical text data is better than that of the other methods in the comparison experiments. CNN is an algorithm that uses convolutional neural networks for relationship classification, while BLSTM has better results for the processing of dynamic sequence problems like text sequences, and the introduction of an attention mechanism based on BLSTM is to enhance the effect of text semantic enhancement and improve the overall effect of the model. The PCNN algorithm in this paper adds location information to the text information and combines word-level attention mechanism and sentence-level attention mechanism of scientific and technical text data. The improvement in accuracy, recall, and F1 value is 2.9%, 1.1%, and 3.6% for the F1 value.

By combining split-word lexicality, bidirectional long- and short-time recurrent network, and attention mechanism, this paper is based on CNN's knowledge graph of speech emotion features to get textual split-words from speech emotion feature data using kipa split-words, then using the split-word vector and character vector representation obtained by Word2vec as the initial input of the model, and the output is the entity vector recognized from speech emotion samples. By combining the subword lexical vectors and introducing the attention mechanism, the entity recognition accuracy of the speech emotion features is improved. For entity-relationship extraction of speech emotion features, an entity-relationship recognition algorithm based on a multiattention mechanism is proposed, while two attention mechanisms, self-attention mechanism and sentence-attention mechanism, are used for entity-relationship classification, and entity-relationship extraction of large data of speech emotion features is realized.

## 4. Conclusion

This paper constructs a knowledge graph of speech emotion features based on impulse-coupled neural networks combined with a generalized corpus database. In the process of word embedding, the pretraining results of the BERT model on a massive text data set are fully utilized to dynamically represent texts with different semantics in different contexts; based on the PCNN model, the feature extraction layer is improved and a multiheaded self-attentiveness mechanism is introduced. In the language recognition model, the results of the model experiments are compared using the control variables method based on a large number of data experiments. In the feature extraction process, different weights are assigned to the words according to their importance to the classification results to enhance the word dependencies within the text; to reduce the limitations of the model in semantic recognition, feature extraction is performed separately in the subspace of multiple feature representations, and then, the results are fused to improve the learning ability of the model. A Chinese named entity recognition model based on the dual-stream self-attentive multidirectional graph is proposed. In the data preprocessing stage, a multidirectional graph method is used to construct the corpus data into the form of a graph, further enrich and optimize the relevant entity dictionaries, and effectively reduce the semantic ambiguity existing in the wordless segmentation of Chinese words. In the text embedding stage, a method based on dynamic weighted fusion is adopted, inspired by the XLNET model. In the text embedding stage, an attention mechanism approach based on dynamic weighted fusion is proposed to improve the semantic understanding of the model.

## Data Availability

The data used to support the findings of this study are available from the author upon request.
